# An mTERF domain protein functions in group II intron splicing in maize chloroplasts

**DOI:** 10.1093/nar/gku112

**Published:** 2014-02-05

**Authors:** Kamel Hammani, Alice Barkan

**Affiliations:** Institute of Molecular Biology, University of Oregon, Eugene, OR 97403, USA

## Abstract

The mitochondrial transcription termination factor (mTERF) proteins are nucleic acid binding proteins characterized by degenerate helical repeats of ∼30 amino acids. Metazoan genomes encode a small family of mTERF proteins whose members influence mitochondrial gene expression and DNA replication. The mTERF family in higher plants consists of roughly 30 members, which localize to mitochondria or chloroplasts. Effects of several mTERF proteins on plant development and physiology have been described, but molecular functions of mTERF proteins in plants are unknown. We show that a maize mTERF protein, Zm-mTERF4, promotes the splicing of group II introns in chloroplasts. Zm-mTERF4 coimmunoprecipitates with many chloroplast introns and the splicing of some of these introns is disrupted even in hypomorphic Zm-*mterf4* mutants. Furthermore, Zm-mTERF4 is found in high molecular weight complexes that include known chloroplast splicing factors. The splicing of two transfer RNAs (*trnI-GAU* and *trnA-UGC*) and one ribosomal protein messenger RNA (*rpl2*) is particularly sensitive to the loss of Zm-mTERF4, accounting for the loss of plastid ribosomes in Zm-*mTERF4* mutants. These findings extend the known functional repertoire of the mTERF family to include group II intron splicing and suggest that a conserved role in chloroplast RNA splicing underlies the physiological defects described for mutations in *BSM/Rugosa2,* the Zm-mTERF4 ortholog in *Arabidopsis*.

## INTRODUCTION

Mitochondria and chloroplasts originated from free-living bacteria and have retained small genomes that primarily encode subunits of their energy-transducing membranes and gene expression machineries. Mechanisms of gene expression in plant organelles combine features acquired from their prokaryotic ancestors with novel features that evolved in their eukaryotic host (1–4). Distinctive features of gene expression in plant organelles include RNA editing, the protein-dependent splicing of group II introns and the processing of messenger RNAs (mRNAs) from longer precursors. The expression and regulation of organellar genes require the participation of hundreds of nuclear-encoded proteins, most of which are unrelated to bacterial proteins or to proteins that act in the nuclear–cytosolic compartment. These features are exemplified by the pentatricopeptide repeat (PPR) family, an unusual family of RNA binding proteins characterized by degenerate helical repeats of ∼35 amino acids (5). PPR proteins bind to specific RNA sequences via a modular 1 repeat-1-nucleotide mechanism (6,7) and participate in various aspects of organelle gene expression that were acquired post-endosymbiosis (8).

The mTERF (mitochondrial transcription termination factor) protein family shares several key features with the PPR family. The mTERF proteins are likewise defined by tandem degenerate helical repeats, although mTERF repeats have only ∼31 amino acids and form three helices instead of two (9,10). Like PPR proteins, mTERF proteins are found only in eucaryotes and almost all are predicted to localize to mitochondria or chloroplasts. In animals, the mTERF family contains three or four members, and these influence mitochondrial transcription, ribosome biogenesis and DNA replication [reviewed in (11)]. By contrast, the genomes of higher plants encode ∼30 mTERF proteins [reviewed in (12)]. Defects in development or stress responses have been linked to mutations in five mTERF genes in *Arabidopsis*, but little information is available concerning the direct molecular functions of the proteins encoded by these genes.

We describe here molecular functions of Zm-mTERF4 (GRMZM2G029933), the maize ortholog of an *Arabidopsis* protein known variously as BSM, RUGOSA2 (RUG2) or mTERF4 (12–14). We show that Zm-mTERF4 is required for the accumulation of plastid ribosomes and for the splicing of several group II introns in chloroplasts. Zm-mTERF4 is found in large complexes in the chloroplast stroma that include intron RNAs and known chloroplast splicing factors, providing strong evidence for a direct role in splicing. Zm-mTERF4 is required for the splicing of several RNAs that are necessary for plastid translation; the failure to splice these introns can account for the loss of plastid ribosomes in Zm-mTERF4 mutants. We suggest that a conserved role in plastid RNA splicing is likely to underlie the developmental and physiological defects described for mutations in *Arabidopsis BSM/RUG2.*

## MATERIALS AND METHODS

### Plant material

A recessive mutation conditioning pale yellow green seedlings and an aberrant population of plastid *atpF* transcripts arose in our collection of *Mu*-transposon-induced non-photosynthetic maize mutants (http://pml.uoregon.edu/photosyntheticml.html). An Illumina-based approach (15) revealed a cosegregating *Mu1* insertion 18-bp upstream of the start codon of locus *GRMZM2G029933*. A second allele was retrieved via a polymerase chain reaction (PCR)-based reverse genetic screen of the same mutant collection; this allele harbors a *MuDR* insertion 10-bp upstream of the start codon that cosegregates with an ivory, seedling lethal phenotype. Complementation crosses involving plants that are heterozygous for each allele produced heteroallelic mutant progeny with an intermediate phenotype, confirming that the chlorophyll deficiency results from disruption of *GRMZM2G029933.* Phenotypically normal siblings of each mutant allele served as the wild-type sample in each experiment. Zm-m*TERF4* is orthologous to *Arabidopsis BSM/RUG2*/At4g02990 (http://cas-pogs.uoregon.edu/#/pog/15427). Other mutants used in this work include *hcf7* mutants, which are pale green due to a global reduction in plastid translation (16) and albino *iojap* mutants, which lack plastid ribosomes entirely (17). Seedlings were grown in soil under 16-h light/8-h dark cycles at 26°C and harvested between 7 and 9 days after planting.

### Generation of anti- Zm-mTERF4 antibodies

The coding sequence of mature Zm-TERF4 (i.e. lacking the transit peptide) lacks introns and was amplified by PCR from *Zea mays* B73 DNA in two steps. First, two overlapping fragments were amplified with primer pairs (i) k134 (5′- GGGGggatccTCCTCCCTCTACGCGCGCCCCAGC) and k137 (5′-GCTCTGTTGTGCAACCAGTTTGTCCCTCAG) and (ii) k136 (5′-CTGAGGGACAAACTGGTTGCACAACAGAGC) and k135 (5′-GGGGaagcttTTATCGAACAAACTCATCATCAGAGTCACC). Second, the two fragments were joined by amplification with primers k134 and k135. The product was digested with BamHI and HindIII and cloned into pMAL-TEV to encode a maltose binding protein–mTERF4 fusion protein. MPB–mTERF4 was expressed in *Escherichia coli*, purified by amylose affinity chromatography and cleaved with TEV protease, and the untagged protein was then purified on a gel filtration column as described previously for HCF107 (18), except that Zm-mTERF4 fractions were subjected to a second amylose affinity chromatography to remove contaminating maltose binding protein. The purified protein was used for immunization of rabbits at Alpha Diagnostic International (http://www.4adi.com/).

### Chloroplast fractionation and protein analysis

Chloroplast subfractions came from a preparation that was described and validated previously (19). Stromal extract was prepared and fractionated as described by Watkins *et al.* (20). Protein–protein coimmunoprecipitation and immunoblot experiments were performed using the antibodies and method described previously (20–25).

### Analysis of RNA

Quantitative reverse transcriptase-polymerase chain reaction (qRT-PCR) was performed as described in (26), using the primers listed in Supplementary Table S1. Unspliced RNA isoforms were amplified with a forward primer complementary to the intron and a reverse primer mapping to the upstream exon. Spliced isoforms were amplified with a forward primer spanning the spliced exons in conjunction with a reverse primer mapping within the upstream exon. RNA gel blot hybridizations were performed as described previously (27).

RNA coimmunoprecipitation assays (RIP-chip) were performed with stromal protein extract and affinity-purified anti-Zm-mTERF4 antibody, using the protocol described in (28). Two RIP-chip experiments were performed using different array designs and independent stromal preparations: The first experiment used the array format described in (29), with overlapping tiled PCR products of ∼500 bp spanning the maize chloroplast genome. The second experiment used custom high resolution microarrays with synthetic 50-mers tiling all annotated maize chloroplast transcripts. The latter microarrays were produced by Mycroarray (http://www.mycroarray.com); the genome coordinates and hybridization signal for each probe are given in Supplementary Table S2. Coimmunoprecipitation with affinity-purified anti-OE16 or anti-AtpB antibody served as the negative control in the first and second RIP-chip experiment, respectively.

## RESULTS

### Recovery of Zm-*mterf4* insertion mutants

**Figure 1.**
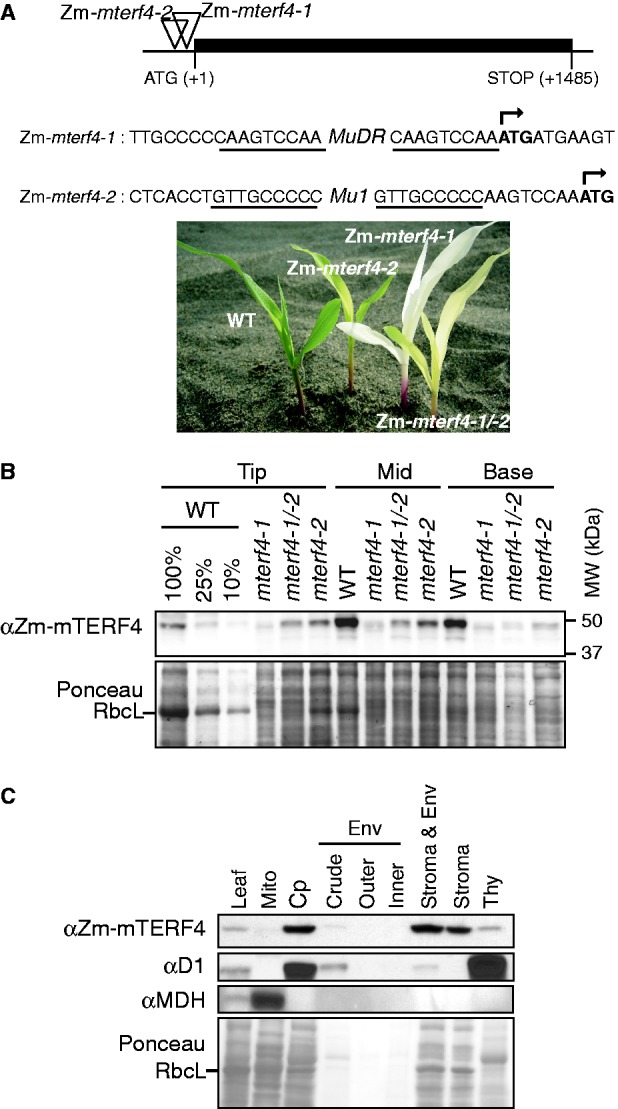
Overview of Zm-*mterf4* mutants. (**A**) Positions of *Mu* transposon insertions in Zm-*mterf4* and the corresponding plant phenotypes*.* The open reading frame, which lacks introns, is indicated by a black rectangle. Sequences flanking the insertions are shown below, with the target site duplications underlined. The start codon is marked with an arrow. Plants were grown in soil for 9 days. The *mterf4-1/-2* plant is the heteroallelic progeny of a complementation cross between Zm-*mterf4-1 and Zm-mterf4-2* heterozygotes. (**B**) Immunoblots showing loss of Zm-mTERF4 in Zm-*mterf4* mutants. Protein was extracted from the base, middle and tip of the second seedling leaf of plants of the indicated genotype. The Ponceau S-stained blot (below) serves as a loading control. The band corresponding to the large subunit of rubisco (RbcL) is marked. (**C**) Subcellular localization of Zm-mTERF4. D1 (PsbA subunit of photosystem II) is a marker for the thylakoid fraction, MDH (malate dehydrogenase) is a marker for the mitochondrial fraction and RbcL shown in the Ponceau-stained blot is a marker for the stromal fraction; these images were reported previously (21) and are reproduced here with permission. Env: envelope; Cp: chloroplasts; Mito: mitochondria; Thy: thylakoid membranes.

Mutant Zm-*mterf4* alleles were recovered during a systematic effort to identify causal mutations in our collection of *Mu* transposon-induced non-photosynthetic maize mutants (30). These mutants came to our attention due to their pale yellow-green phenotype (Figure 1A) and their aberrant population of transcripts from the plastid *atpF* gene (see later in the text)*. Mu* insertions that cosegregate with this phenotype were sought with an Illumina-based method for sequencing the DNA flanking all *Mu* insertions in individual plants (15). Among the several cosegregating insertions that were identified, an insertion in GRMZM2G029933 stood out as a good candidate for the causal mutation because its gene product is orthologous to *Arabidopsis* At4g02990 (known also as *BSM, RUG2* and *mTERF4),* whose mutation disrupts chloroplast biogenesis (13,14). We assigned the name Zm-*mterf4* to this gene, in accordance with a nomenclature proposed recently for the *Arabidopsis* mTERF family (12). Zm-mTERF4 is predicted to harbor nine consecutive mTERF repeats. A structural model generated by I-TASSER (31) predicts that these helical repeats continue outside of the nine predicted mTERF motifs and make up virtually the entirety of the protein other than a predicted chloroplast targeting sequence at the N-terminus (Supplementary Figure S1).

An independent insertion in the same gene was recovered in a reverse genetic screen of our mutant collection and cosegregates with an ivory seedling phenotype (Figure 1A). Complementation crosses between plants heterozygous for each allele yielded ∼25% chlorophyll-deficient heteroallelic progeny with an intermediate phenotype (Figure 1A), confirming that the chlorophyll deficiency results from disruption of Zm-*mterf4*. The severity of the visual phenotypes is consistent with the positions of the insertions: the Zm-*mterf4-1* allele harbors an insertion 10-bp upstream of the predicted start codon and conditions an ivory leaf phenotype, whereas the Zm-*mterf4-2* insertion lays 18-bp upstream of the predicted start codon and conditions a pale green phenotype. Plants that are homozygous for either allele die after the development of three to four leaves upon exhaustion of seed reserves, as is typical for non-photosynthetic maize mutants.

### Zm-mTERF4 localizes to the chloroplast stroma

A polyclonal antibody raised against recombinant Zm-mTERF4 was used to probe immunoblots of proteins from basal, middle and apical sections of the second seedling leaf, which contain chloroplasts at increasing stages of differentiation (32). The antibody detected a protein running slightly ahead of the 50 kDa marker, consistent with the size of mature Zm-mTERF4 (49 kDa) (Figure 1B). The abundance of this protein was strongly diminished in the basal and middle leaf sections of Zm-*mterf4* mutants, confirming it to be Zm-mTERF4. The magnitude of the Zm-mTERF4 protein deficiency correlated with that of the chlorophyll deficiency in plants harboring each combination of mutant alleles. Zm-mTERF4 protein was detectable even in plants homozygous for the stronger allele, Zm-*mterf4-1*. Zm-mTERF4 builds up to ∼50% of normal levels at the tip of Zm-*mterf4-2* mutant leaves. An analogous developmental profile was observed for the chloroplast splicing factor RNC1 in hypomorphic *rnc1* mutants (20). These results suggest that a reduced rate of Zm-mTERF4 and RNC1 synthesis in hypomorphic mutants is compensated by increased protein stability as chloroplast development proceeds.

To determine the intracellular location of Zm-mTERF4, we probed immunoblots of leaf, chloroplast and mitochondrial extracts with the Zm-mTERF4 antibody (Figure 1C). The results show that Zm-mTERF4 localizes to chloroplasts, consistent with its detection in the maize plastid nucleoid proteome (33); the results suggest further that Zm-mTERF4 is absent from mitochondria. Localization of Zm-mTERF4 to chloroplasts and not to mitochondria is consistent with findings of Babiychuk *et al.* (13) for the *Arabidopsis* ortholog BSM/RUG2. Thus, it is possible that the dual-localization of a BSM/RUG2-GFP fusion protein to both chloroplasts and mitochondria (14) was an artifact of overexpression. Analysis of chloroplast subfractions show that Zm-mTERF4 is found primarily in the stromal fraction.

### Zm-mTERF4 is required for the accumulation of plastid ribosomes

**Figure 2.**
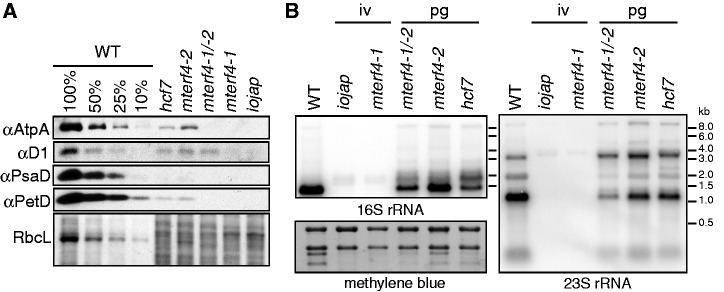
Loss of plastid ribosomes and proteins in Zm-*mterf4* mutants. (**A**) Immunoblots of leaf extract (5 µg protein and the corresponding dilutions) were probed with antibodies against core subunits of photosynthetic enzyme complexes: AtpA (ATP synthase), D1 (photosystem II), PsaD (photosystem I) and PetD (cytochrome *b_6_f*). The Ponceau S-stained blot is shown below as a loading control and demonstrates the abundance of RbcL, the large subunit of rubisco. (B) Total seedling leaf RNA (1 µg) was analyzed by RNA gel blot hybridization using probes for the indicated chloroplast rRNAs. The methylene blue stained blot that was probed for 16 S rRNA is shown to illustrate equal loading of cytosolic rRNAs (top two bands). The visible phenotypes of the mutant plants used for RNA extraction are indicated above: pg: pale green; iv: ivory.

Maize mutants with albino phenotypes similar to that conditioned by the Zm-*mterf4-1* allele typically have severe plastid ribosome deficiencies (e.g.^19–21^,^34^). Core subunits of each photosynthetic enzyme complex harboring a plastid-encoded subunit [adenosine triphosphate (ATP) synthase, photosystem II, photosystem I, cytochrome *b_6_f* and rubisco] are severely reduced even in hypomorphic Zm-*mterf4-2* and Zm-*mterf4-1/-2* mutants (Figure 2A). Furthermore, Zm-*mterf4* mutants have reduced levels of the plastid 16S and 23S ribosomal RNAs (rRNAs), and the magnitude of the rRNA deficiency corresponds with the degree of chlorophyll deficiency (Figure 2B). Therefore, Zm-mTERF4 is required for the accumulation of plastid ribosomes, as is *BSM* in *Arabidopsis* (13).

### Zm-mTERF4 associates with chloroplast group II introns and stimulates splicing *in vivo*

**Figure 3.**
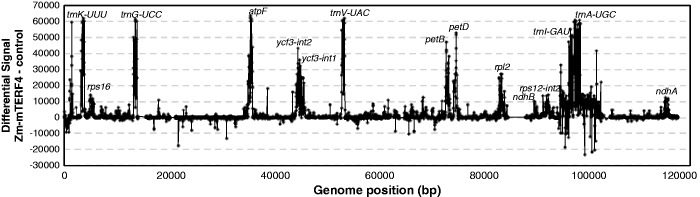
RIP-chip analysis to identify RNA ligands of Zm-mTERF4. Stromal extract was subjected to immunoprecipitation with the Zm-mTERF4 antibody or with antibody to AtpB (a subunit of the chloroplast ATP synthase) as a negative control. RNAs from the immunoprecipitation pellets were labeled with Cy3 fluorescent dye and hybridized to a high resolution oligonucleotide tiling microarray of the maize chloroplast genome. The signal in the Zm-mTERF4 pellet fraction is plotted after subtracting the corresponding signal in the AtpB control. The genomic region harboring each group II intron is marked. The peaks are largely confined to intron sequences, although exons are coimmunoprecipitated in some cases. A bipartite peak maps within the *trnK* intron; the valley corresponds to the intron-internal *matK* open reading frame. The fluorescence values for each genome coordinate can be viewed in Supplementary Table S2. A replicate dataset using a different array design and independent stromal preparation is shown in Supplementary Figure S2.

The plastid ribosome deficiency conditioned by mutations in Zm-*mterf4* together with the *atpF* RNA defect that drew our attention to these mutants suggested that Zm-mTERF4 influences plastid gene expression at the post-transcriptional level. To determine whether Zm-mTERF4 associates with chloroplast RNA *in vivo*, we used genome-wide RNA coimmunoprecipitation assays (29). Stromal extract was subject to immunoprecipitation with affinity-purified Zm-mTERF4 antibody, and coimmunoprecipitating RNAs were identified by hybridization to tiling microarrays of the maize chloroplast genome. Replicate assays performed with different stromal preparations and different microarray formats gave similar results (Figure 3 and Supplementary Figure S2). Several RNAs were strongly enriched in the Zm-mTERF4 coimmunoprecipitation, all of which harbor group II introns. Enrichment was particularly apparent for intron sequences from *trnK-UUU, trnG-UCC, atpF, ycf3, trnV-UAC, petB, petD, rpl2, trnI-GAU* and *trnA-UGC*.

To address whether Zm-mTERF4 influences the splicing of the introns with which it associates, the ratio of spliced to unspliced RNA for each plastid group II intron was assessed in Zm-*mterf4* mutants. Because plastid ribosome deficiency causes pleiotropic effects on the splicing of plastid introns (35), splicing in Zm-*mterf4* mutants was compared with that in other maize mutants with ribosome deficiencies of a similar magnitude: pale green Zm-*mterf4-2* and Zm-*mterf4-1/-2* mutants were compared with pale green *hcf7* mutants, which exhibit a moderate loss of plastid ribosomes (16); albino Zm-*mterf4-1* mutants were compared with albino *iojap* mutants, which lack plastid ribosomes entirely (17). The relative abundance of plastid rRNAs in these mutants can be seen in Figure 2B.

**Figure 4.**
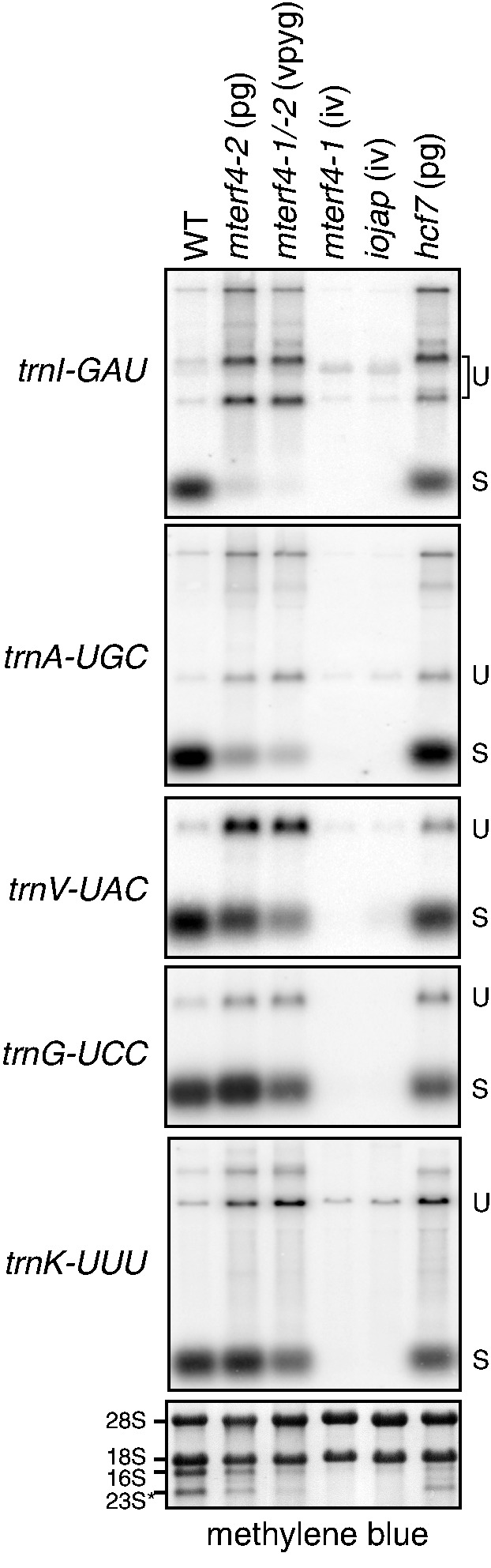
RNA gel blot hybridizations demonstrating chloroplast tRNA splicing defects in Zm-*mterf4* mutants. Seedling leaf RNA (1 µg) was analyzed by probing with exon sequences from the indicated tRNA genes. Unspliced (U) precursors and spliced tRNAs (S) are marked. The visible phenotype of each mutant is indicated: pg: pale green; iv: ivory. Blots were stained with methylene blue to ensure equal loading of cytosolic RNAs (18S and 28S), and a representative image is shown below. The stained bands corresponding to plastid rRNAs (16S and 23S*, an *in vivo* fragment of 23S rRNA) illustrate the ribosome deficiency in the mutants.

The splicing of plastid transfer RNAs (tRNAs) was assayed by RNA gel blot hybridization using exon probes (Figure 4). The accumulation of spliced *trnI-GAU* and *trnA-UGC* was severely compromised even in the hypomorphic Zm-*mterf4-2* and Zm-*mterf4-1/-2* mutants but was only minimally reduced in *hcf7* mutants. The effect on *trnV*-UAC was slightly more severe in Zm-*mterf4* mutants than in the corresponding controls (as reflected by the higher ratio of unspliced to spliced RNA), whereas *trnK-UUU* and *trnG-UCC* were spliced to a similar extent in the Zm-*mterf4* and control mutants. These results provide strong evidence that Zm-mTERF4 is required for the splicing of *trnI-GAU* and *trnA-UGC*, and they suggest that Zm-mTERF4 may stimulate trnV-UAC splicing. The results are consistent with a role for Zm-mTERF4 in the splicing of other tRNAs harboring group II introns, but these splicing defects may instead be a consequence of the loss of plastid ribosomes in Zm-*mterf4* mutants.

**Figure 5.**
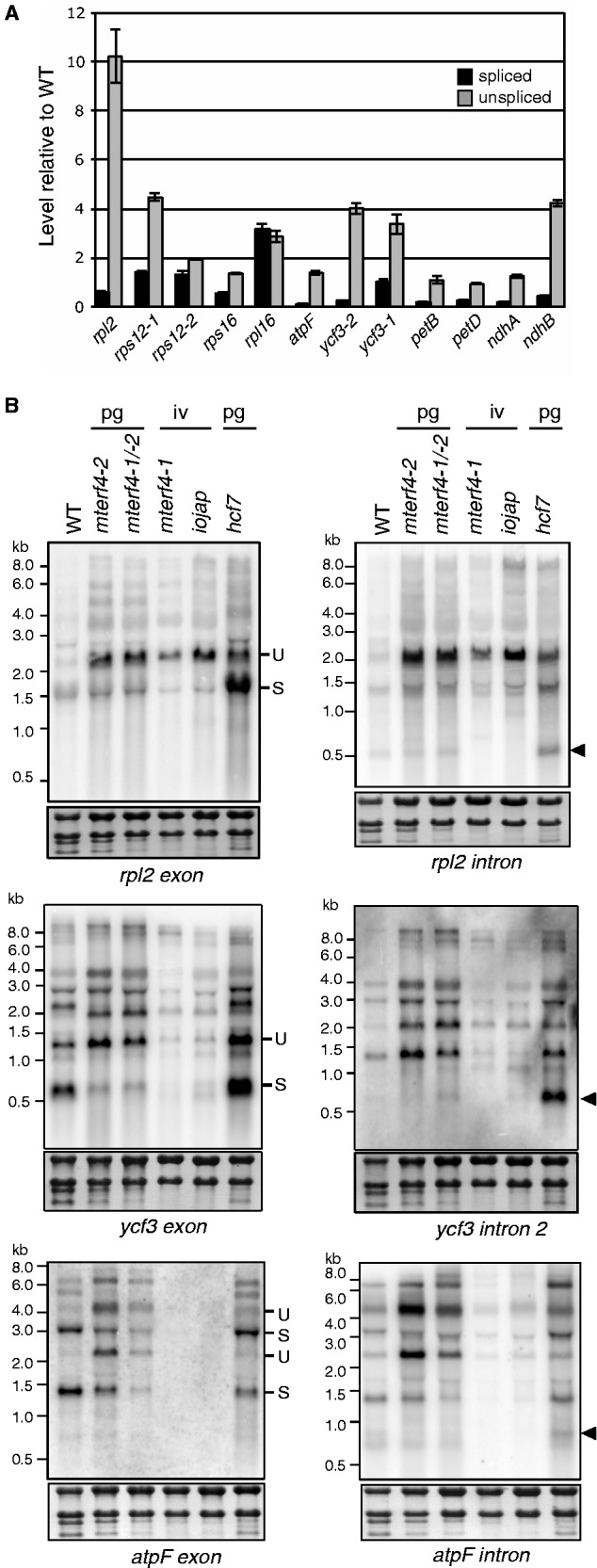
Defects in chloroplast pre-mRNA splicing in Zm-*mterf4* mutants. (**A**) qRT-PCR assays of spliced and unspliced chloroplast mRNAs in *mterf4-1/-2* mutants*.* Seedling leaf RNA was analyzed by qRT-PCR using two sets of primers: one set specifically amplified spliced RNA by using a primer spanning spliced exons; the other set specifically amplified unspliced RNA by using one exon and one intron primer. Values represent transcript abundance relative to that in wild-type (WT) siblings and are means of two technical replicates. (**B**) RNA gel blot analyses illustrating defects in pre-mRNA splicing in Zm-*mterf4* mutants. Seedling leaf RNA (3 µg) from plants of the indicated genotype was hybridized with the indicated probes. The visible phenotype of each mutant is indicated above: pg: pale green; iv: ivory. The membranes were stained with methylene blue and excerpts harboring the rRNA bands are shown at bottom. Arrows mark transcripts that we believe to represent excised introns based on their hybridization to intron probes and their apparent size. Examples of spliced (S) and unspliced (U) transcripts are marked. These results demonstrate a role for Zm-mTERF4 in the splicing of the *atpF*, *rpl2* and *ycf3-2* introns, as Zm-*mterf4* mutants exhibit an increased ratio of unspliced RNA to each product of the splicing reaction (spliced RNA and excised intron), in comparison with control mutants with equivalent losses of plastid ribosomes.

A qRT-PCR assay (Figure 5A) revealed an increased ratio of unspliced to spliced RNA for most intron-containing plastid mRNAs in hypomorphic Zm-*mterf4-1/-2* mutants. However, defects in the splicing of *rpl2*, *ndhB*, *atpF* and *ycf3-*intron 2 appeared to be particularly severe, whereas the splicing of *rps12, rps16* and *rpl16* pre-mRNAs was minimally affected. RNA gel blot hybridizations confirmed that hypomorphic Zm-*mterf4* alleles condition stronger defects in the splicing of the *atpF*, *rpl2* and *ycf3-2* introns than do corresponding control mutants (Figure 5B), that *rps12, rpl16* and *rps16* splicing is not significantly disrupted (Supplementary Figure S3), and that *petB* and *petD* splicing is slightly reduced (Supplementary Figure S3). However, the RNA gel blot and qRT-PCR data were inconsistent in the case of the *ndhB* intron: whereas qRT-PCR indicated a strong splicing defect, the RNA gel blot data showed little, if any, defect (Supplementary Figure S3). We do not have an explanation for this difference, but the clarity of the RNA gel blot data argue against a role for Zm-*mterf4* in *ndhB* splicing.

Taken together, these results separate introns into three categories. (i) The *trnI, trnA, rpl2, atpF* and *ycf3-2* introns coimmunoprecipitate with Zm-mTERF4, and their splicing is strongly enhanced by Zm-mTERF4*.* (ii) The *ndhA*, *rps12, rpl16, rps16* and *ndhB* introns are only weakly enriched in Zm-mTERF4 coimmunoprecipitations, and these introns do not require Zm-mTERF4 for their splicing. (iii) The *trnK, trnG, trnV, ycf3-1, petB* and *petD* introns are strongly enriched in Zm-mTERF4 coimmunoprecipitations, but splicing defects in Zm-*mterf4* mutants were either minor or difficult to evaluate due to secondary effects resulting from the loss of plastid ribosomes.

### Zm-mTERF4 is found in large complexes and is associated with known splicing factors *in vivo*

**Figure 6.**
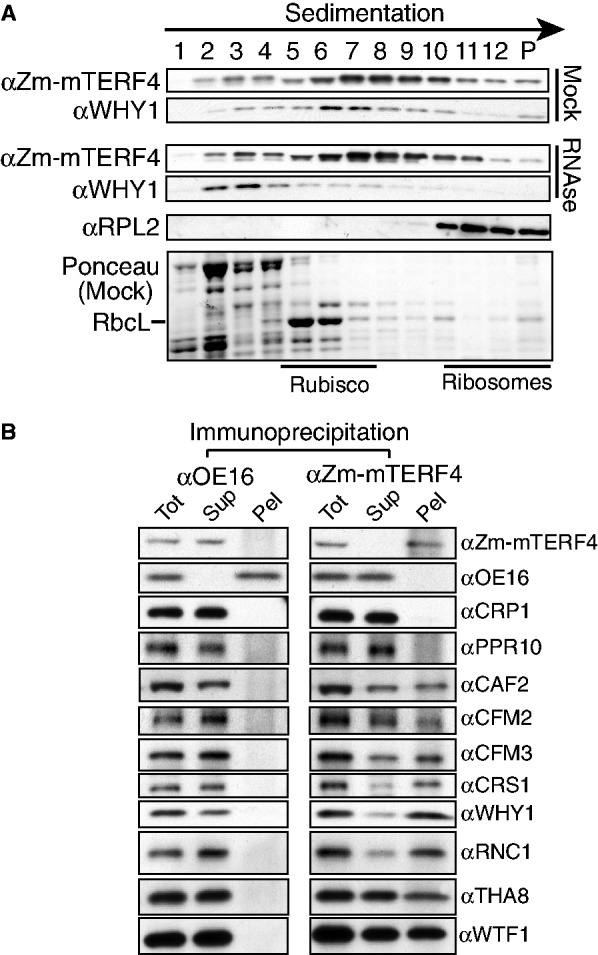
Zm-mTERF4 is found in high molecular weight complexes *in vivo* and associates with known chloroplast splicing factors. (**A**) Sucrose gradient fractionation of mock-treated and RNase-A-treated stroma. An equal volume of each fraction was analyzed on immunoblots by probing with antibodies indicated at left. Zm-WHY1 is a multifunctional protein that binds to both RNA and DNA and promotes the splicing of the *atpF* intron (25); it is included here to show that the ribonuclease treatment was successful. RPL2 is a protein in the plastid large ribosomal subunit. The Ponceau S-stained blot of the mock-treated stromal fractions is shown below. (**B**) Zm-mTERF4 coimmunoprecipitates with chloroplast splicing factors. Stromal extract was immunoprecipitated with Zm-mTERF4 antibody. An immunoprecipitation with OE16 antibody served as a negative control. One-tenth of the immunoprecipitation pellets (Pel) or supernatants (Sup) and one-twentieth of the input (Tot) were analyzed on immunoblots by probing with antibodies indicated at right. CAF2, CFM2, CFM3, CRS1, WHY1, RNC1, THA8 and WTF1 are chloroplast group II intron splicing factors (20,21,23,25,36–39). CRP1 and PPR10 associate with specific chloroplast mRNAs (24,29) but are not involved in splicing.

Many previously identified group II intron splicing factors in chloroplasts reside in large intron-containing complexes that sediment in a broad peak between rubisco (∼550 kDa) and ribosomes (∼1 MDa) (20,21,23,36–38). Analysis of sucrose gradient-fractionated chloroplast stroma showed that Zm-mTERF4 is found in particles in this same size range (Figure 6A). Ribonuclease treatment of stroma before sucrose gradient centrifugation did not reduce the presence of Zm-mTERF4 in high molecular weight particles. This has also been observed for several other chloroplast group II intron splicing factors (25,37,38) and is likely due to the compact tertiary structure of group II introns and to the fact that plastid introns are tightly associated with many proteins.

To determine whether Zm-mTERF4 is found in particles containing previously identified chloroplast splicing factors, Zm-mTERF4 was immunoprecipitated from chloroplast stroma, and the pellet and supernatant fractions were analyzed by probing immunoblots with antibodies to various chloroplast RNA binding proteins (Figure 6B). The chloroplast splicing factors CAF2, CFM2, CFM3, CRS1, WHY1, RNC1, THA8 and WTF1 coimmunoprecipitate with Zm-mTERF4, consistent with the fact that these proteins share intron ligands with Zm-mTERF4 [see summary of intron ligands in (23)]. In contrast, PPR10 and CRP1, which bind specific non-intronic regions on plastid mRNAs (24,29), do not coimmunoprecipitate with Zm-mTERF4. In conjunction with the RIP-chip and sedimentation data, these results provide strong evidence that Zm-mTERF4 is found in intron-containing complexes that include other group II intron splicing factors.

## DISCUSSION

Results presented here extend the known functional repertoire of the mTERF protein family by demonstrating a role for Zm-mTERF4 in group II intron splicing. We show that Zm-mTERF4 associates *in vivo* with many chloroplast introns and splicing factors*,* and that it is required for the efficient splicing of several of the introns with which it associates. This role in plastid RNA splicing impacts the biogenesis of the plastid translation machinery, which in turn affects photosynthesis and plant viability.

Molecular functions for only a handful of mTERF proteins have been established. The mTERF proteins in metazoa affect mitochondrial transcription, DNA replication and translation [reviewed in (11,40)], and several of these functions are understood in considerable mechanistic detail. For example, human MTERF1 binds to a specific site on mitochondrial DNA and triggers transcription termination by acting as a ‘roadblock’ via a mechanism that involves flipping several bases away from the DNA duplex (9,41,42). Some mTERF proteins in invertebrates [reviewed in (39)] and in the alga *Chlamydomonas reinhardtii* (43,44) likewise function in mitochondrial transcription termination, suggesting this to be an ancient function of the mTERF family. However, the potential for mTERF motifs to mediate diverse macromolecular interactions became clear with the recent discovery of two mammalian mTERF proteins that function in the biogenesis of mitochondrial ribosomes. A human protein denoted MTERF4 (not orthologous to the subject of this article) recruits an rRNA methyl transferase to the mitochondrial large ribosomal subunit; one end of the mTERF α-solenoid binds to the rRNA methyltransferase, and a positively charged surface along the length of the solenoid was proposed to bind RNA (45–47). A metazoan protein denoted MTERF3 is likewise involved in the biogenesis of mitochondrial ribosomes, although its precise role is unknown; importantly, this protein was shown to interact with rRNA *in vitro* (48), providing the first direct evidence that mTERF repeats can interact with RNA. Our results add a new example of an RNA-related function for an mTERF protein. Although our attempts to assay nucleic acid binding activity by recombinant Zm-mTERF4 have produced inconsistent results (data not shown), the coimmunoprecipitation of Zm-mTERF4 with intron RNAs, the splicing defects in Zm-*mterf4* mutants and the fact that one metazoan mTERF protein has been shown to bind RNA, suggest that Zm-mTERF4 interacts directly with intron RNA. It will be interesting to discover the structural variations within mTERF repeat tracts that confer RNA *versus* DNA binding activity.

The mTERF family in plants is considerably larger than that in metazoa, comprising roughly 30 members; all of these are predicted or demonstrated to localize to mitochondria or chloroplasts (reviewed in 12). The few plant mTERF proteins that have been studied emerged in screens for mutations that disrupt abiotic stress responses or embryogenesis (12–14,49–52), but the direct molecular functions underlying these effects are not known. Our finding that Zm-mTERF4 is a chloroplast splicing factor is likely to be relevant to other plant mTERF proteins. In fact, our results were foreshadowed by evidence that BSM, the ortholog of Zm-mTERF4 in *Arabidopsis*, is required to splice intron 2 in the chloroplast *clpP* pre-mRNA (13), a group II intron that is not found in maize. Defects in *atpF, rpl2* and *rps12-*intron 2 splicing were also detected in that study. However, it was not clear whether these splicing defects were a direct or indirect effect of the mutation, as these introns fail to splice in the absence of plastid translation (35,53) and *bsm* mutant plastids lack ribosomes. Although Zm-*mterf4* mutants are likewise deficient for plastid ribosomes, we were able to distinguish direct effects on splicing from indirect effects due to compromised plastid translation by (i) comparing plastid splicing in hypomorphic mutants to that in other mutants with ribosome deficiencies of a similar magnitude and (ii) performing coimmunoprecipitation assays to detect physical associations between Zm-mTERF4, intron RNAs and known plastid splicing factors. Our results suggest that Zm-mTERF4 functions directly in *atpF* and *rpl2* splicing but does not play an important (direct) role in *rps12-*intron 2 splicing. Neither BSM nor Zm-mTERF4 is required for the *trans-*splicing of *rps12-* intron 1. The splicing of other introns was not assayed in the *bsm* mutant, but it seems likely that the molecular functions described here are conserved among monocots and dicots, as has been shown for many other chloroplast splicing factors (23,36,37,54–56). As such, it can be anticipated that the *trnI*, *trnA* and *ycf3-2* introns, which strongly coimmunoprecipitate with Zm-mTERF4 and whose splicing is particularly sensitive to its loss, are likewise direct targets of BSM/RUG2. A direct role for Zm-mTERF4/BSM/RUG2 in *rpl2*, *trnI* and *trnA* splicing is sufficient to account for the loss of plastid ribosomes in the maize and *Arabidopsis* mutants. However, the downstream effects of severe plastid ribosome deficiency differ in *Arabidopsis* and maize: the maize mutants germinate to yield albino seedlings that die after the development of ∼3 leaves, whereas the *Arabidopsis* mutants fail to complete embryogenesis. Analogous observations involving other orthologous mutants in maize and *Arabidopsis* have been made previously (e.g. 23,34,54,57) and are believed to be due to differences in plastid gene content in the two species [reviewed in (52)].

Sixteen nucleus-encoded proteins had previously been demonstrated to participate in the splicing of the ∼20 group II introns in land plant chloroplasts [reviewed in (58)]. These proteins are found in large ribonucleoprotein complexes together with their intron ligands, and they function combinatorially to promote the splicing of various intron subsets. Most such proteins belong to RNA binding protein families that are dedicated to organellar gene expression and that appear to have evolved in that context; these include the CRM (59), PPR (60), APO (56) and PORR (21) domain families. Zm-mTERF4 now adds another example. As CRM, PPR and PORR domain proteins also function in group II intron splicing in plant mitochondria (61–63), it seems likely that additional group II intron splicing factors will be discovered among mitochondrial mTERF proteins.

Zm-mTERF4 coimmunoprecipitates with many known chloroplast splicing factors, and it is found in stromal particles of a similar size. The contribution of protein–protein *versus* protein–RNA interactions to Zm-mTERF4’s *in vivo* intron specificity cannot be determined at this time. However, Zm-mTERF4 is more promiscuous than previously described chloroplast splicing factors in that it associates with many introns *in vivo* and it does not have a strong effect on the splicing of all of the introns with which it associates. The fact that Zm-mTERF4 associates with some introns without strongly impacting their splicing suggests one of two possibilities: either it is functionally redundant with a different protein or these interactions are opportunistic with no functional significance. The former possibility is consistent with the highly similar RIP-chip profiles for Zm-mTERF4 and the PORR domain splicing factor WTF1 (21). The latter possibility is interesting from an evolutionary perspective, as opportunistic interactions between weakly specific RNA binding proteins may provide a reservoir from which obligate interactions can subsequently evolve.

The mTERF family joins the PPR and OPR families as organelle-dedicated nucleic acid binding families with a helical repeat architecture (59,64,65). It seems that the tandem repeat architecture may be a particularly amenable scaffold for the evolution of new nucleic acid binding functions in the organellar context. Many mTERF proteins were detected in the maize chloroplast nucleoid proteome, a major site of plastid RNA and DNA metabolism (33). In addition, many mTERF proteins in *Arabidopsis* are coexpressed with proteins known to be involved in organellar gene expression (12). Thus, the elucidation of the functional repertoire—both molecular and physiological—of the numerous mTERF proteins in plants will likely lead to further insights into the expression and evolution of organellar genomes.

## SUPPLEMENTARY DATA

Supplementary Data are available at NAR Online.

## FUNDING

Postdoctoral fellowship from the European Molecular Biology Organization and a Marie Curie grant [CIG-618492-plantMTERF to K.H.]; US National Science Foundation [IOS-0922560 to A.B.]. Funding for open access charge: National Science Foundation [IOS-0922560].

*Conflict of interest statement*. None declared.

## Supplementary Material

Supplementary Data
