# Supplementary material for: An mTERF domain protein functions in group II intron splicing in maize chloroplasts
Source: Nucleic Acids Res. 2014 Feb 5;42(8):5033–42. doi: 10.1093/nar/gku112 (PMC4005652; doi:10.1093/nar/gku112)
Supplement: Supplementary Data [file supp_42_8_5033__index.html]

An mTERF domain protein functions in group II intron splicing in maize chloroplasts — An mTERF domain protein functions in group II intron splicing in maize chloroplasts — Supplementary Data 

# An mTERF domain protein functions in group II intron splicing in maize chloroplasts

## Supplementary Data

files

**Files in this Data Supplement:**

- Supplementary Data - pdf file
- Supplementary Data - xlsx file
