# Supplementary material for: An mTERF domain protein functions in group II intron splicing in maize chloroplasts
Source: Nucleic Acids Res. 2014 Feb 5;42(8):5033–42. doi: 10.1093/nar/gku112 (PMC4005652; doi:10.1093/nar/gku112)

Supplementary Table S1

Supplementary Table S1: List and sequences of oligonucleotides used for qRT-PCR.

**Chloroplastic Genes**	**Forward Primer:**	**Sequence (5'>3'):**	**Reverse Primer:**	**Sequence (5'>3'):**
rps12-int1 spliced	spliced rps12-int1_F1	GGAACATGTGCTAGGGTGTATACTA	spliced rps12-int1_R1	AATGCGATATCTCACACCGGGT
rps12-int1 unspliced	rps12-int1_F2	TGGATTTGCACCAAAGGAAACCA	rps12-int1_R2	GCCGCGCTTAAGGGATGTCC
ycf3-int2 spliced	spliced ycf3-int2_F1	GAATGGCCTGTTCTCCTCGGTAA	spliced ycf3-int2_R3	GCGACTAGAAATTGATCCCTATGAT
ycf3-int2 unspliced	ycf3-int2_F2	TCCTGGAGTAAGCGCTATAGCTTGT	ycf3-int2-R2	AGCAATTTCTGAGCCGTATGAGGT
petD spliced	spliced petD_F1	AAGGCGGATTATGGGAGTAACA	spliced petD_R1	CACCAATCATTGACGGCTCT
petD unspliced	petD_F2	TGTCCGGTTCCTTTGGGGGATGG	petD_R2	AAAGATCGTTGGGCCACGCGG
atpF spliced	spliced atpF_F1	GATTTTTTTTGGAAAGGGAGTGTTAAA	spliced atpF_R1	TACTCATCTGCTTCCAGTTCGAC
atpF unspliced	atpF_F2	AGCGGGAGAGCCAAATGAATCGA	atpF_R2	AGGGTCCCTTTACGCAATTCTTCCG
ndhA-spliced	spliced ndhA_F3	CCTGCTACTAATTCTTCTTCTGCTT	spliced ndhA_R3	GTGTACTAGCAATATCTCTACTATC
ndhA-unspliced	ndhA_F2	AGGCTGACGCCAAAGATTCCATCC	ndhA_R2	AGAGGAGCCGTATGAAGCTAAGGTT
rpl2 spliced	spliced rpl2_F1	GGGAAATGCCCTACCTTTGACCG	spliced rpl2_R1	CCAAACGGACCTCCCCAGATGGT
rpl2 unspliced	rpl2_F2	TCAATGGGAAATGCCCTACCTTTG	rpl2_R2	TGAACTCAATCACTTGCTGCCGT
petB spliced	E250petBf-2	GTTTTGGTATCTCTGGAATATGAGTAAAGTA	E249 petBr	TATACTCGAGCTATAACGGACCCGAAATACCTT
petB unspliced	petB_F4	ACCGTGGCTGATGGAGTTTTGAACC	petB_R4	CTGCAATTGCCTGAATCTCAAGACG
ndhB spliced	E248 ndhB_F	CTCCTGACGTCTACGAAGGATCCC	E248 ndhB_R	GCCCTATGGACGAATATGCAAGCAT
ndhB unspliced	ndhB_F3	AGCAACGACTGGAGTGGGGGA	ndhB_R3	TCACTTAGGAGCCGTGCGAGA
rps12-int2 unspliced	rps12-int2_F1	TGGTAGCCTGCTCCAGTCCCC	rps12-int2_R1	CGAGGAACCCTAGATGCTGTCGC
rps12-int2 spliced	spliced rps12-int2F	GCCAGAGTACGATTAACCTCTGGAT	spliced rps12-int2R	CATATTTAGAACGCCCTTGTTGACGAT
ycf3-int1 unspliced	ycf3-int1F1	CTCACCTTCCCCAAGCGCGG	ycf2-int1R1	TCCGACAACCTCCGGGGAAA
ycf3-int1 spliced	spliced ycf3_int1F	GGGCATTTACTTATTATAGAGATGGGATG	spliced ycf3_int1R	AGCCTTTGTATGCTCTCCATTGCTT
rpl16 unspliced	rpl16F1	TGCCTCGGCAGGATTTTCCCT	rpl16R1	TCATTCGCGAGGAGCTGGATGA
rpl16 spliced	spliced rpl16_F	ATGCTTAGTCCCAAAAGAACTAGATTTCG	spliced rpl16_R	CGTGTCATTGCTCTTCGTCCTGC
rps16 unspliced	rps16_F1	CGAAGATCTCTTCCTTCTCTTCGAG	rps16_R1	TCGAGCCGTATGAGGAGAAAACCT
rps16 spliced	spliced rps16_F	CGATGTGGTAGAAAGCAACAAGCTATC	spliced rps16_R	AGGTTGAGCACCCTTTTCAAGGA
**Nuclear Reference Gene**	**Forward Primer:**	**Sequence (5'>3'):**	**Reverse Primer:**	**Sequence (5'>3'):**
high mobility group GRMZM2G024976	zmHMG_F1	AAAGGGGCATCAACACATCTTCA	zmHMG_R1	CGGAGTTGGCAAAGCAGGTTCT
high mobility group GRMZM2G024976	zmHMG_F2	CTAAGTCCTGAAGATAAAGCTGTA	zmHMG_R2	AGCAGCATATCATCTTGGCTC

Supplementary Figure S1. Predicted structure of Zm-mTERF4. This model was generated by I-TASSER (http://zhanglab.ccmb.med.umich.edu/I-TASSER/). It begins at amino acid 58 (the predicted transit peptide cleavage site) and ends at the stop codon. The arrows mark the boundaries of the mTERF repeat tract as redicted by InterPro. Each mTERF motif is colored differently to demarcate the boundaries of the repeats. The ten best templates used by I-TASSER to generate this model were PDB entries 3n6sA, 3n7qA, 3v6pA, 3ugmA, 3v6tA, 3m66A, 2z5nA, 3opgA, 2zxqA, and 1qgkA.

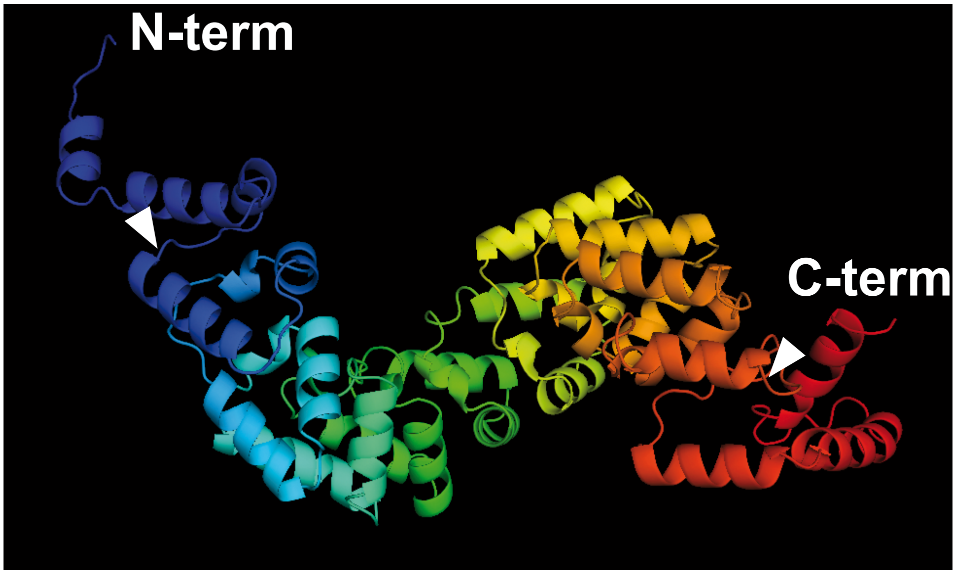

Supplementary Figure S2. Replicate RIP-chip assay

Stromal extract was subjected to immunoprecipitation with the Zm-mTERF4 antibody. RNAs from the pellet and supernatant were differentially labeled with fluorescent dyes and hybridized to a tiling microarray of the maize chloroplast genome harboring overlapping PCR products of ~500 bp. The $\log_2$ transformed enrichment ratio (Pellet/Supernatant) was plotted according to chromosomal position, after subtracting the $\log_2$ ratios obtained from a control immunoprecipitation using antibody to OE16. Array elements harboring group II introns are marked.

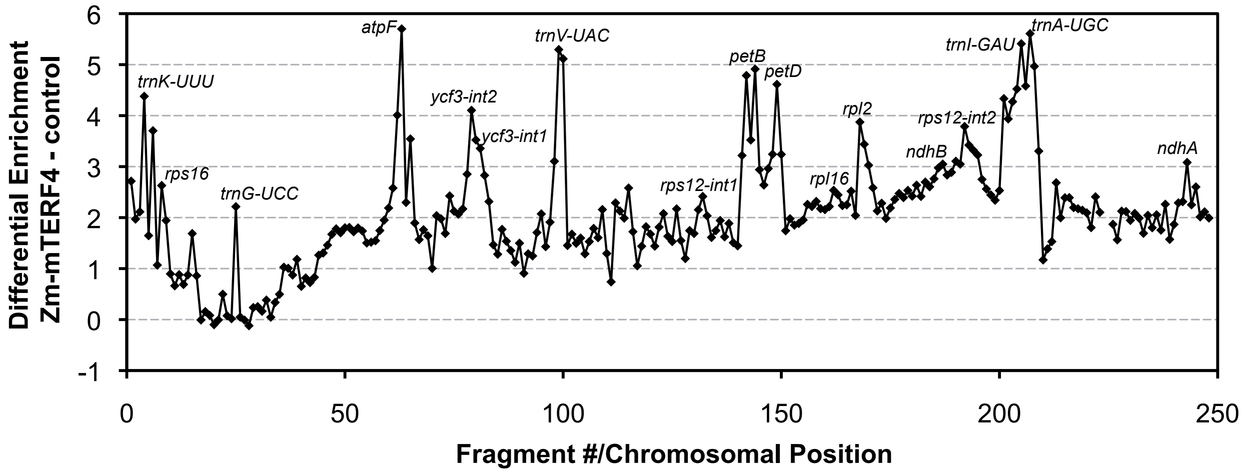

Supplementary Figure S3. Additional analyses of chloroplast intron splicing in *Zm-mterf4* mutants by RNA gel blot hybridization. 3 μg of seedling leaf RNA from plants of the indicated genotype were hybridized with the indicated probes. The visible phenotype of each mutant is indicated above: pg -pale green; iv - ivory. The membranes were stained with methylene blue and excerpts harboring the rRNA bands are shown at bottom. Arrows mark bands that we believe to represent excised introns based on their hybridization to intron probes and their apparent size. *tha8* mutants are pale green and non-photosynthetic due to defects in the splicing of *ycf3*-intron 2 and *trnA* (23). Arrows mark bands that we believe to be excised introns based on their apparent size.

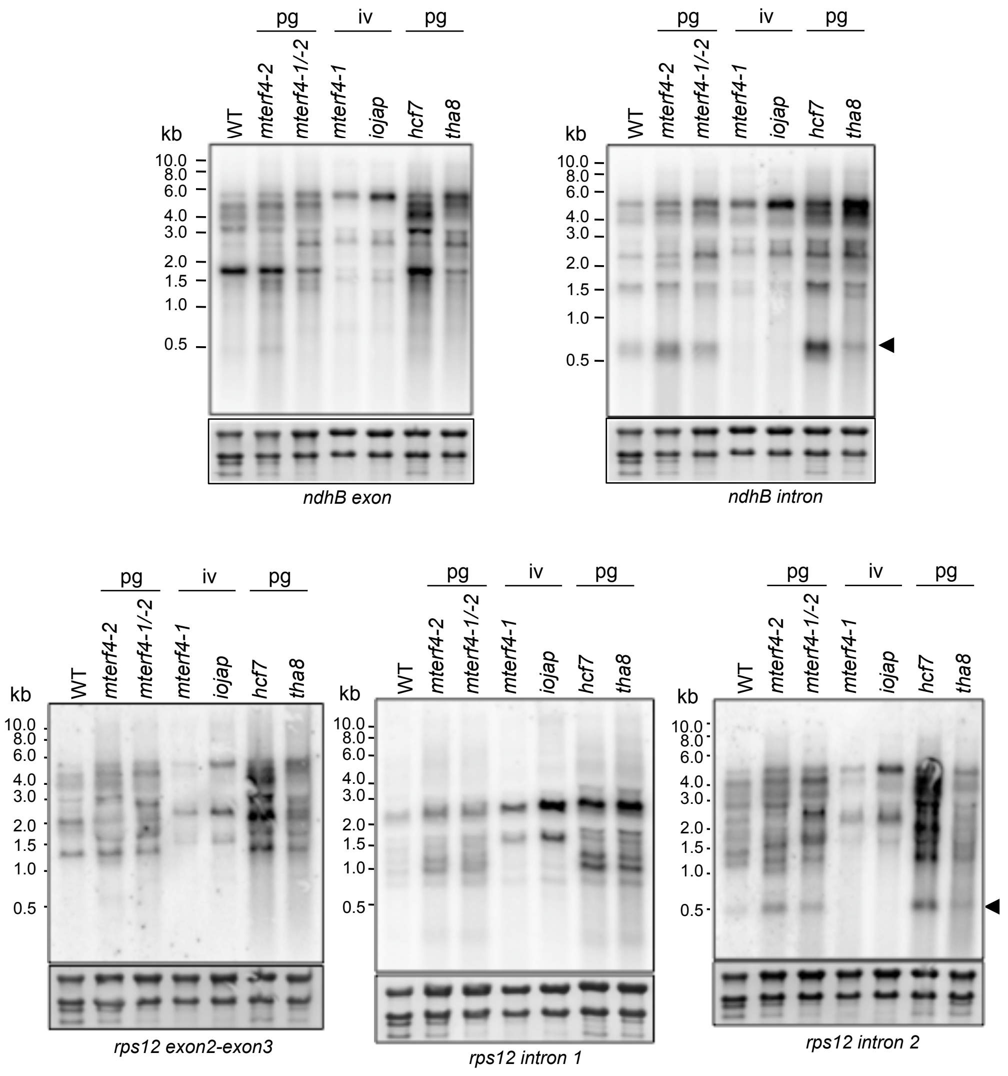

Supplementary Figure S3 continued

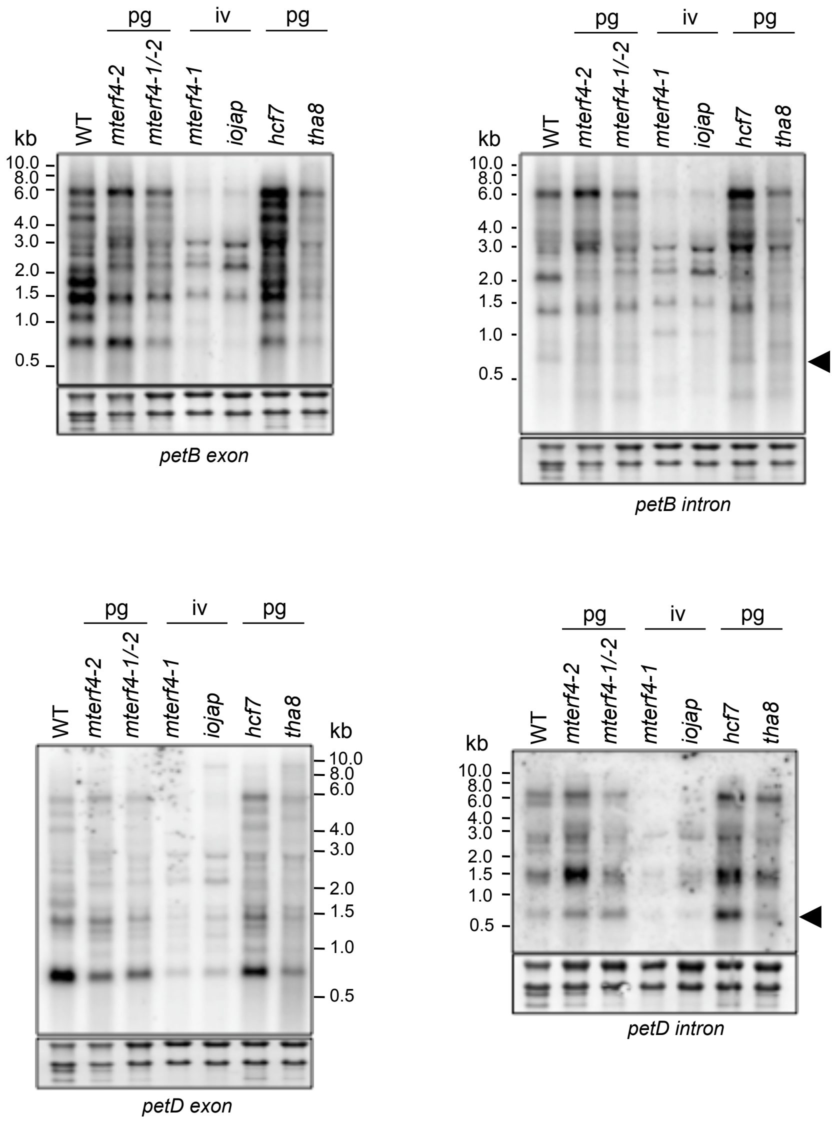

Supplementary Figure S3 continued

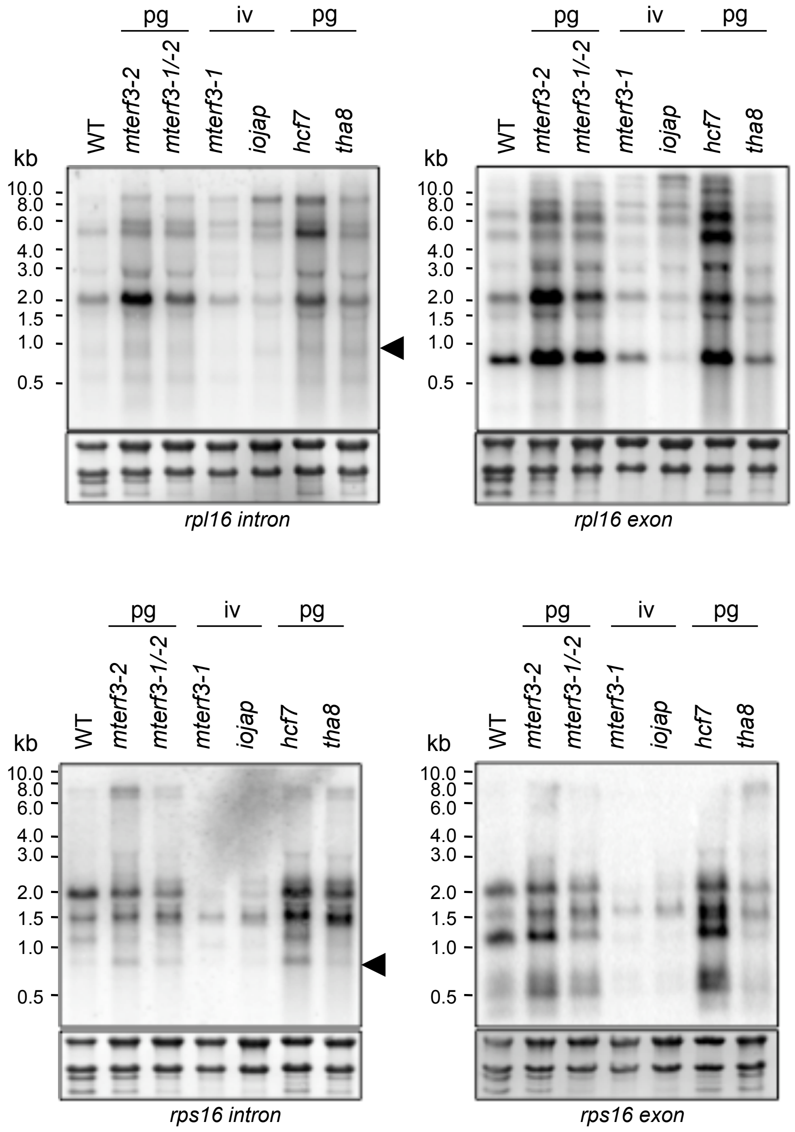

Supplementary Figure S3, continued

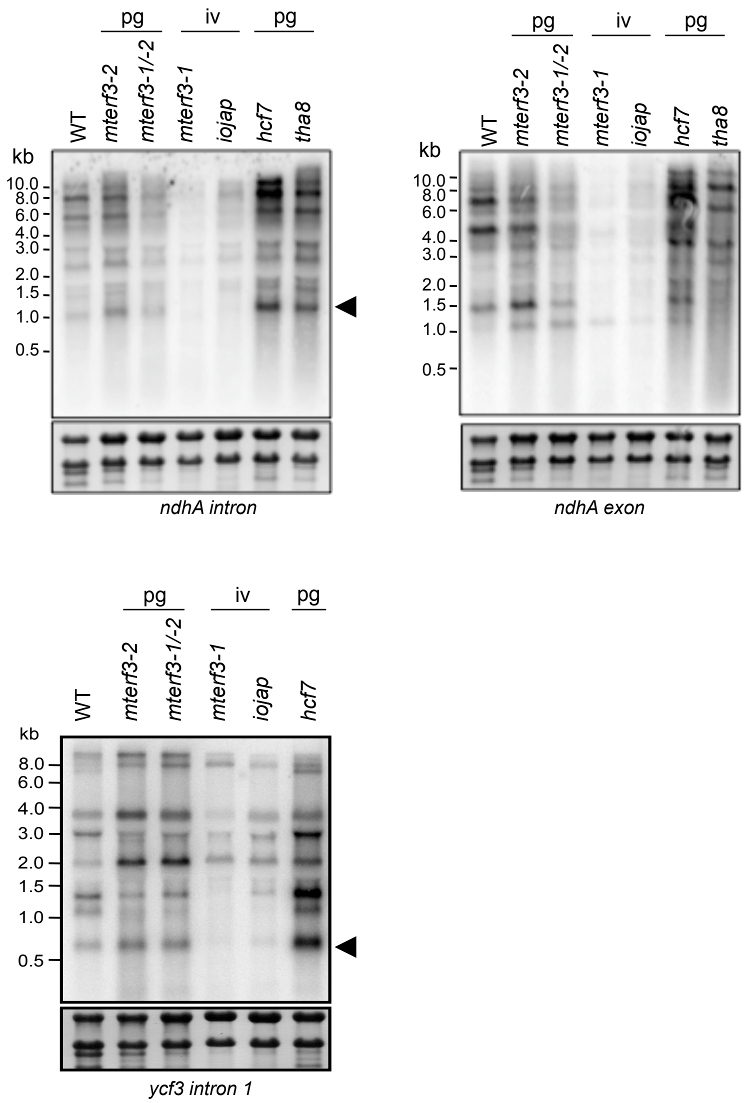

Supplement: Supplementary Data [file supp_gku112_nar-01606-a-2013-File008.pdf]
